# Long-Term Isothermal Phase Transformation in Lead Zirconate

**DOI:** 10.3390/ma15124077

**Published:** 2022-06-08

**Authors:** Dariusz Kajewski, Irena Jankowska-Sumara, Jae-Hyeon Ko, Jeong Woo Lee, Syed Furqan Ul Hassan Naqvi, Rafał Sitko, Andrzej Majchrowski, Krystian Roleder

**Affiliations:** 1 Institute of Physics, University of Silesia, ul. 75 Pułku Piechoty 1, 41-500 Chorzów, Poland; krystian.roleder@us.edu.pl; 2 Institute of Physics, Pedagogical University, ul. Podchorążych 2, 30-084 Kraków, Poland; irena.jankowska-sumara@up.krakow.pl; 3 School of Nano Convergence Technology, 1 Hallymdaehakgil, Chuncheon 24252, Gangwondo, Korea; hwangko1@gmail.com (J.-H.K.); jwlee6209@gmail.com (J.W.L.); furqanhassan05@gmail.com (S.F.U.H.N.); 4 Institute of Chemistry, University of Silesia, ul. Szkolna 9, 40-006 Katowice, Poland; rafal.sitko@us.edu.pl; 5 Institute of Applied Physics, Military University of Technology, ul. gen. Sylwestra Kaliskiego 22, 00-908 Warsaw, Poland; andrzej.majchrowski@wat.edu.pl

**Keywords:** lead zirconate, phase transition, Raman scattering

## Abstract

Lead zirconate PbZrO_3_ has been the subject of research interest for several dozen years. Recently, even its antiferroelectric properties have started to be questioned, and many researchers still deal with the so-called intermediate phase below Curie temperature (T_C_), whose existence is not fully understood. It turns out that PbZrO_3_ doped with Nb exhibits below T_C_ phases with complex domain structures. One of them undergoes self-organization taking place at a constant temperature, and transforms, after several minutes, into a lower phase. This isothermal transition was investigated through dielectric, pyroelectric current and Raman scattering measurements. Discontinuities accompanied it in the permittivity and pyroelectric current. The obtained Raman spectra proved that those discontinuities are strictly linked with the isothermal transition between two intermediate phases. The ordering process in lead sublattice stimulated by thermal fluctuations is discussed as a driving force for this peculiar phenomenon.

## 1. Introduction

Structural phase transitions in the so-called functional materials have been intensively studied for dozens of years. In order to understand the mechanisms leading to their appearance, when relating them to temperature, one modifies e.g., by pressure, light, electric or magnetic fields. The causes of phase transitions observed in one of the most thoroughly studied compounds, namely, lead zirconate (PbZrO_3_, PZO), remain controversial and disputable [1,2,3,4,5,6,7,8,9,10,11,12,13,14,15]. Ceaselessly, the occurrence of an intermediate phase, separating the paraelectric phase from the antiferroelectric phase, is considered. It has been theoretically discussed in papers [15] in which trilinear coupling in free energy expansion has been introduced. It was shown that under some conditions of this coupling, an energetically available intermediate polar phase may appear. The authors of Ref. [16] determined—through x-ray diffraction—two groups of crystals that differ in phases’ symmetry. Namely, the group with no intermediate phase presents an almost perfect perovskite structure opposite to that demonstrating such a phase. Therefore, doping (or admixtures) and intentionally defected crystal [10] extends the temperature range in which the intermediate phase develops. Moreover, the PZO crystal doped with heterovalent ions, such as niobium [17,18,19,20,21,22], causes the appearance of a second intermediate phase, not observed before [21,22,23]. In addition, a substantial temperature hysteresis of phase existence was observed in that crystal [20,21]. It should be remembered that the difference between the free energy per unit cell for ferroelectric and antiferroelectric phases is of the order of 1 meV only. Recently, phase evolution triggered by the illuminating electron beam has been observed for PZO crystals with atomic resolution [24]. Utilizing the negative spherical-aberration imaging technique, quantitative transmission electron microscopy (TEM) reveals a hierarchical evolution of the polar oxygen octahedra and successive polarisation rotation during the stepwise antiferroelectric-ferroelectric phase transition. In particular, it has been found that the antiferroelectric–ferroelectric transition is initiated by the emergence of a novel ferroelectric category, a ferrodistortive phase, which is characteristic of antiparallel Pb displacements and particular cycloidal order of polarisation [22]. Moreover, another phenomenon may play an essential role in relaxation processes in the phase of co-existing antiferroelectric and ferroelectric states. This is the flexoelectricity, manifesting by a sharp flexo-coupling peak at the T_C_ of PZO [23]. Therefore, all that mentioned above points to the non-trivial co-existence of metastable states below T_C_ in this material. If so, the stability of physical properties over time, especially in such metastable phases, cannot be ruled out. Indeed, the isothermal phase transition observed in PZO ceramics subjected to pressure [25] and PZT single crystals [26,27,28] supports it.

In most cases, the phase transition is independent of time, being only a function of temperature. The transition usually occurs in an extremely short time of 10^−12^–10^−9^ s [29,30], much faster than the experimentally available cooling speed. The temperature change modifies the differences in free energy between the high and low symmetry phases, which consequently causes a transition.

Unlike normal time-independent phase transitions, a different type of transition may occur in isothermal conditions like standard pressure and specific temperatures. This transition progresses over time and takes place after several minutes and even hours. This type of transition is known as the isothermal phase transition.

Isothermal phase transitions were observed in various compounds with perovskite structure, such as BaTiO_3_: Ni, Cr [31], Na_0.5_B_0.5_TiO_3_ (NBT) [32], K (Ta, Nb) O_3_ (KTN) [33], Pb (Zr, Ti) O_3_ (PZT) [27,28] and other K_2_ZnCl [34] and LiNH_4_C1_4_ [35]. Only a small group of materials was mentioned here, in which this phenomenon was observed, but so far, no systematic research related to this phenomenon has been undertaken. There are only different types of hypotheses that have not gained the status of isothermal phase transition theory in ABO_3_ materials.

In the case of NBT, the isothermal phase transition was explained through the coexistence of polar regions in the paraelectric matrix of the crystal lattice. These regions are meant to stabilize and begin to interact [36]. In the case of ceramics (1−x)Bi_0.5_Na_0.5_TiO_3_−xBaTiO_3_, an isothermal transition was postulated as being realized through a metastable state, which is a thermodynamically unbalanced phase between two stable phases [37]. In these two examples, the cause of isothermal phase transitions is perceived completely differently. However, in both cases, the source of the existence of polar regions and the reasons for their expansion at constant temperature has not been shown.

The first observations of the isothermal phase transition in PZO ceramics were made utilizing dielectric tests [26]. The measuring furnace was heated up to the set temperature and left until the thermal equilibrium was achieved after about 4 min. The observed time changes indicated that there was no complete phase transition between the two phases at a given temperature. At that time, it was only suggested that this process occurs through the nucleation of regions with a different symmetry, but the authors did not indicate the cause of this nucleation.

Nearly 50 years later, research on the BNT-BT system [36], and the isothermal phase transition observed in it, presents a phenomenological model based on the Landau theory of free energy. Free energy changes, and more precisely, the transition of the system between one minimum and the other minimum, are to be caused by point defects or impurities creating randomly distributed fields of local polarization and breaking the symmetry, which will ultimately lead to the formation of long-range ordered domains. An appropriately high concentration of defects conditions the isothermal transition. An important fact is that these studies were based, like 50 years earlier, only on the observation of dielectric properties.

Hence, we have undertaken studies of time-dependent permittivity, pyroelectric current and Raman scattering for PZO single crystals with small niobium content to determine whether these time-dependent effects are intrinsic or extrinsic. In particular, these studies answered the question concerning the nature of phase transition between two intermediate phases appearing in doped PZO crystals.

## 2. Materials and Methods

Nb-doped PZO single crystals with a nominal concentration of 0.2 and 1.0 mol% Nb_2_O_5_ were grown from high-temperature solutions using spontaneous crystallization (flux growth). The details of the growth procedure for the nominal concentrations 0.2 mol% Nb_2_O_5_ and 1.0 mol% Nb_2_O_5_ have been published in [18,20], and [22], respectively.

The Nb content in crystals was analyzed using energy-dispersive x-ray fluorescence (EDXRF) spectrometer—Epsilon 3 (Panalytical, Almelo, The Netherlands) with an Rh target X-ray tube operated at the maximum voltage of 30 keV and a maximum power of 9 W. The spectrometer has been equipped with a thermoelectrically cooled silicon drift detector (SDD) with an 8 μm Be window and a resolution of 135 eV at 5.9 keV. Quantitative analysis was performed using Omnian software based on the fundamental parameter method and the following measurement conditions: 30 kV, 300 µA, 120 s counting time, air atmosphere and 100 μm Ag primary beam filter. The Nb content on crystals was equal to 0.0772 ± 0.0037 and 0.107 ± 0.0087 mol%, respectively. Further in the text, the content 0.077 and 0.1 mol% of Nb are used. It has to be mentioned that both crystals were grown using different procedures. Since the phase transition sequence observed in these crystals was the same, we state that their properties do not depend on crystal technology.

An automated measuring system with Agilent 4192A impedance analyzer was used to measure the capacity C and conductance G in parallel circuit mode. Keithley 6514 electrometer was used for pyroelectric and thermally stimulated depolarization current measurements. Before performing the measurements, silver paste electrodes were attached to the samples and rejuvenated at 390 °C for 10 min.

Micro-Raman backscattering measurements were performed at the wavelength of 532 nm by using a conventional Raman spectrometer LabRam HR800, Horiba Co. combined with a compact temperature controller (THMS600, Linkam). An optical microscope (BX41, Olympus) was used for backscattering geometry. Using a holographic notch filter, it was possible to measure the Raman spectrum down to 10 cm^−1^. A single grating with 1800 lines/mm was used, resulting in a resolution of 0.5 cm^−1^.

## 3. Results

### 3.1. Dielectric Characteristics and Pyroelectric Current Measurements of PbZrO_3_:0.077Nb Crystal

**Figure 1:**
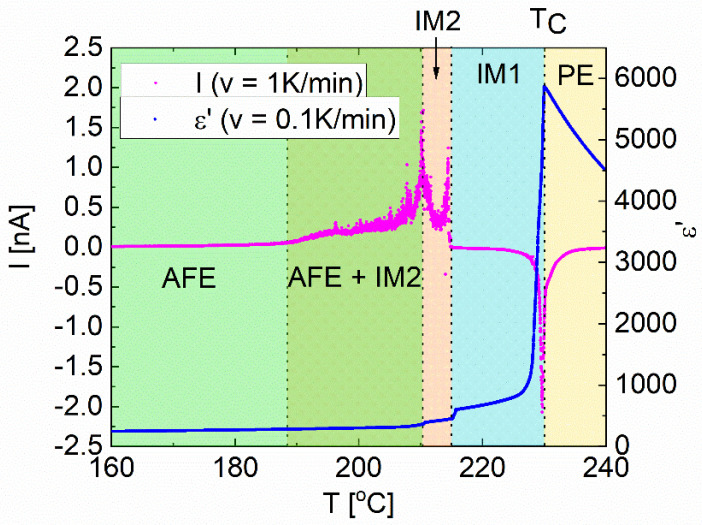
Temperature-dependent permittivity (in blue) and total (primary and secondary) pyroelectric current (in pink) in PbZrO_3_:0.077Nb crystal.

The dielectric properties and pyroelectric current values were measured on cooling, with temperature rates of 0.1 K/min and 1 K/min, respectively. In both experiments, three distinct anomalies were observed (Figure 1). The first was connected with the paraelectric (PE) to intermediate transition (IM1) at T_C_. The second was between intermediate phases IM1 and IM2. The third anomaly was associated with the transition to antiferroelectric phase AFE with polar regions immersed in its structure. This phase co-existence was within the 20 °C range, and thus, the pyroelectric current was easily observed (Figure 1). It was detected down to the temperature of about 190 °C due to gradually disappearing polar regions in the antiferroelectric matrix [21,22]. Therefore, the existence of two intermediate phases and the co-existence of phases have been proven. Similar behaviour was noted in previously investigated PZO crystals doped with Nb [21,23].

**Figure 2:**
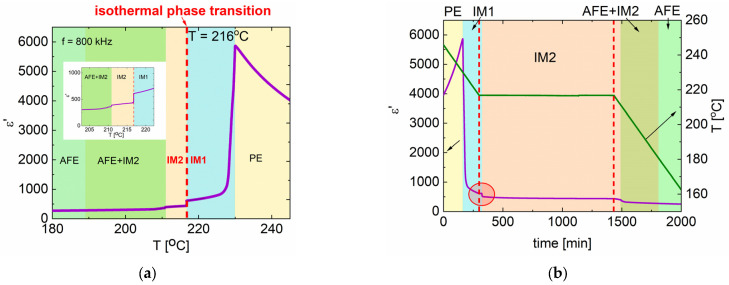

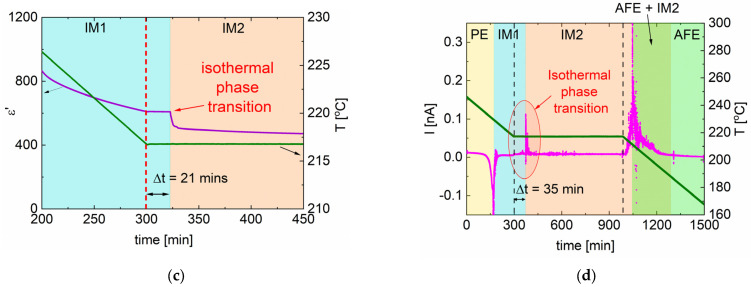
(**a**) Temperature changes of permittivity—the red dashed line marks the temperature at which time-dependent measurements were performed and presented in (**b**,**c**). (**b**) Permittivity and temperature as a function of time—the red dashed lines represent the time when the temperature was stopped at 216 °C—the red circle region is presented in (**c**). (**c**) Permittivity changes in the vicinity of the isothermal M1–M2 transition are marked as a red circle in (**b**). (**d**) Total pyroelectric current and temperature as a function of time for PbZrO_3_:0.077Nb crystal single crystal (the red circle marks the isothermal phase transition).

Because of the substantial temperature hysteresis of phase transitions observed in these crystals [20,21], we have decided to examine the time stability of the first intermediate phase (IM1). Due to the readily discernible transition between the first and second intermediate phases, the IM1 phase was selected for experiments. The results of dielectric and pyroelectric measurements are presented in Figure 2a–d, respectively. The procedure was to stop the cooling below T_C_ but above the transition between the IM1 and IM2 phases. In the case of PbZrO_3_:0.077Nb transition point was at 214.5 °C (Figure 1), and so the observation of the dielectric and pyroelectric properties in time was performed at 216 °C (Figure 2a). In the case of permittivity, the step-like jump was observed after 21 min of temperature stabilization. It corresponded to the IM1–IM2 transition (Figure 2b). After that jump, the permittivity exponentially decreased (Figure 2c). About 500 min later, the cooling was started again, and eventually, the phase transition from IM2 to AFE phase took place (Figure 2a,b).

Similar observations were made by measuring the total pyroelectric current (Figure 2d). After about 35 min at 216 °C, a sharp increase in pyroelectric current was observed, followed by current sparks resembling the Barkhausen effect. As in the case of permittivity measurements, cooling was resumed 510 min later, and a further increase in pyroelectric current was observed at 215 °C. This transition is related to the IM2–AFE matrix phase transition.

The difference in times after the isothermal phase transition began can undoubtedly be explained in several ways. The different volumes of co-existing domains and time needed for defects to be charged neutralized, domains wall distributions, orientations and sizes of domains growing below T_C_, have to be considered. All these influence local strains and, thus, the relaxation process. However, further studies of this problem should be performed to know which of the mentioned phenomena play an essential role. For example, this could be done through time-dependent neutron scattering studies or pressure-dependent Brillouin light scattering. Yet, a theoretical model of isothermal transformation would help find a convincing explanation.

As can be seen, the anomalies of permittivity and pyroelectric current connected with the IM2–AFE transition demonstrate the exact nature, irrespective of whether the measurements were performed at temperature changes or in time but at a constant temperature. It means that the IM2–AFE transition can also be isothermal.

### 3.2. Raman Light Scattering in PbZrO_3_:0.1Nb Crystal

To better understand the isothermal transition mechanism, Raman light scattering measurements were performed as a function of time. For these experiments, a PZO crystal with 0.1 mol% Nb was chosen. This choice was made because the temperature dependence of Raman light scattering for this crystal was carefully investigated and reported in ref. [23]. The measurement procedure of Raman light scattering in time was the same as that applied to the dielectric and pyroelectric current measurements described above. The crystal was first heated to 350 °C to reach a pure paraelectric state and then cooled to 300 °C. Raman light scattering was measured from 300 °C to 233 °C. At 233 °C, cooling was stopped, and measurements were performed in time inside the IM1 phase. The data were collected every 2 min for 50 min, and then the cooling was started again. The spectra obtained as a function of temperature were the same as those presented in [23], i.e., above the T_C_; the first-order Raman spectra induced by local symmetry break were observed [23,38,39,40].

**Figure 3:**
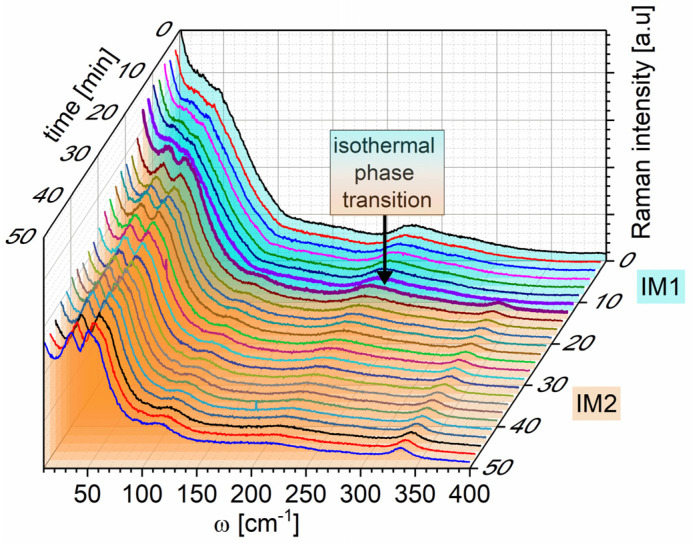
Time-dependent evolution of Raman spectra for PbZrO_3_:0.1Nb single crystal at 233 °C. The line colors are only used to make it easier to distinguish spectra obtained at different times.

At T_C_ = 238 °C, a change in the Raman spectra was observed associated with the transition to the IM1 phase. Maintaining constant temperature at 233 °C, we discovered that, after about 14 min, a further evolution of the spectrum occurred, finally assuming the shape characteristic for the IM2 phase [23] (Figure 3). It means that the isothermal IM1–IM2 transition occurs.

The analysis of recorded spectra was performed using the damped harmonic oscillator model for phonon modes, the same used in [23]. In Ref [23] also, a detailed fitting methodology was presented. The model used assumes the response of one relaxor (quasi-elastic or Rayleigh part of the spectrum) and several damped harmonic oscillators (inelastic part of the scattering) [41]. Thus, the intensity *I_R_* of the spectrum as a function of frequency for a given temperature/time can be described as:

(1) $$I_{R}(\omega )=\left\{\begin{array}{c} n(\omega )+1\\ n(\omega ) \end{array}\right\}\left(\frac{S_{r}\gamma_{r}\omega }{\omega^{2}+\gamma_{r}^{2}}+\sum_{i}\frac{S_{i}\omega_{i}^{2}\gamma_{i}\omega }{{\left(\omega_{i}^{2}-\omega^{2}\right)}^{2}+\gamma_{i}^{2}\omega^{2}}\right)$$

where *S_i_, ω_i_, γ_i_* are the strength, frequency and damping factor of the ith vibration mode, respectively, while *S_r_* and *γ_r_* are the strength and level of relaxation of the relaxor, respectively. The coefficients *n(ω)* + 1 and *n(ω)* are Bose–Einstein coefficients that refer to Stokes and anti-Stokes scattering, described by the formula:

(2) $$\left[n\left(\omega \right)+1\right]={\left[1-\exp\left(\frac{\hbar \omega }{k_{B}T}\right)\right]}^{-1}$$

To compare the spectra obtained, the Bose–Einstein population factor was used:

(3) $$I_{R}\left(\nu \right)=I_{obs}\left(\nu \right)\left[1-\exp\left(-\frac{hc\nu }{k_{B}T}\right)\right]\frac{\nu \nu_{0}^{3}}{{\left(\nu_{0}-\nu \right)}^{4}}$$

where *I_obs_* and *I_R_* are observed and reduced Raman intensities, respectively; *ν* and *ν_0_* are the Raman shift and the wavenumber of the excitation source, respectively. The *h*, *k_B_*, *c*, and *T* represent, respectively, Planck’s constant, Boltzmann’s constant, the speed of light and temperature.

**Figure 4:**
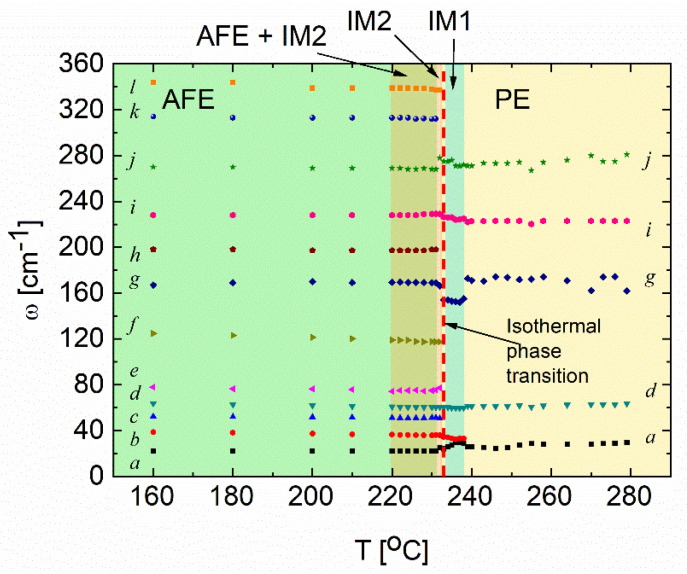
Temperature dependence of Raman frequencies for PbZrO_3_:0.1Nb crystal. The dotted line corresponds to the isothermal transition point (the red dashed line points to the 233 °C at which the time evolution is presented in Figure 5). The colored regions correspond to transitions at T_PE-IM1_ and T_IM2-AFE_ detected in the permittivity and optical measurements [23].

By fitting this model to the recorded spectra, we determined the frequency *ω_i_*, damping factor *γ_i_* and the vibration force *S_i_* as a function of temperature and time. Temperature changes in frequencies of the observed vibration modes are presented in Figure 4. They correspond to the vibrations described in [22]. The modes 250–360 cm^−1^ originate from Zr-O torsional modes. Especially modes near 350 cm^−1^ originated from the AFE character of PbZrO_3_ [42]. Modes between 150 and 250 cm^−1^ are the so-called torsional modes, whereas the lowest frequency region 10–150 cm^−1^ is related to the so-called external or lattice modes. Hence, it is sensitive to crystal structure and phase transitions. Among them, the mode near 120 cm^−1^ is a well-known antiferrodistorsive mode connected with octahedral tilts and assigned as a soft mode in lead zirconate and compounds on its basis [15].

**Figure 5:**
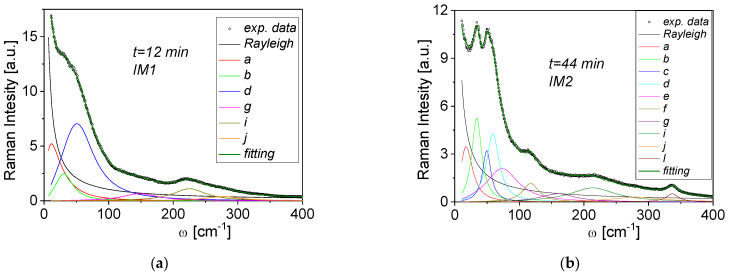
Examples of Raman spectra fitting correspond on a timescale to the (**a**) IM1 phase and (**b**) IM2 phase.

**Figure 6:**
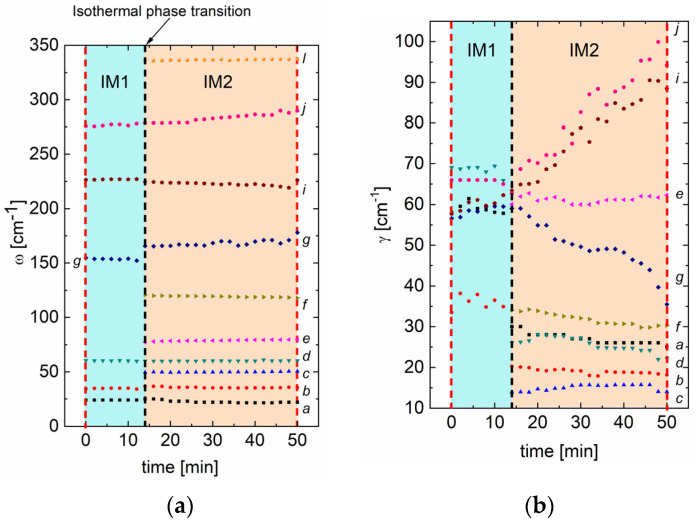
(**a**) Raman frequency and (**b**) damping factor of calculated modes, as a function of time for PbZrO3:0.1Nb crystal at 233 °C. The black dashed line corresponds to the isothermal phase transition. The red dashed lines correspond to the time span of the measurement at 233 °C in Figure 4.

As mentioned before, the spectrum evolution in time was measured at 233 °C (see the dotted line in Figure 4). After about 14 min, vibration modes associated with the IM2 phase appeared (Figure 5a). Two representative fittings for the spectra from IM1 and IM2 phases are presented in Figure 5a,b. Figure 6 represents the temperature dependences of mode frequencies below 400 cm^−1^ obtained from the fitting. Phase changes are visible as sudden frequency changes and the disappearance or appearance of some modes. Moreover, discontinuities at the IM1–IM2 transition was observed for both factors. At the same time, no significant changes in the frequency (Figure 6a) and no significant changes in the damping factor of modes inside the IM1 phase were observed (Figure 6b). It follows that the phase transformation is purely structural and of the 1st order. The question remains about the exact structure and symmetry of the IM1 phase. Current research on neutron scattering should provide a solution to the puzzle.

**Figure 7:**
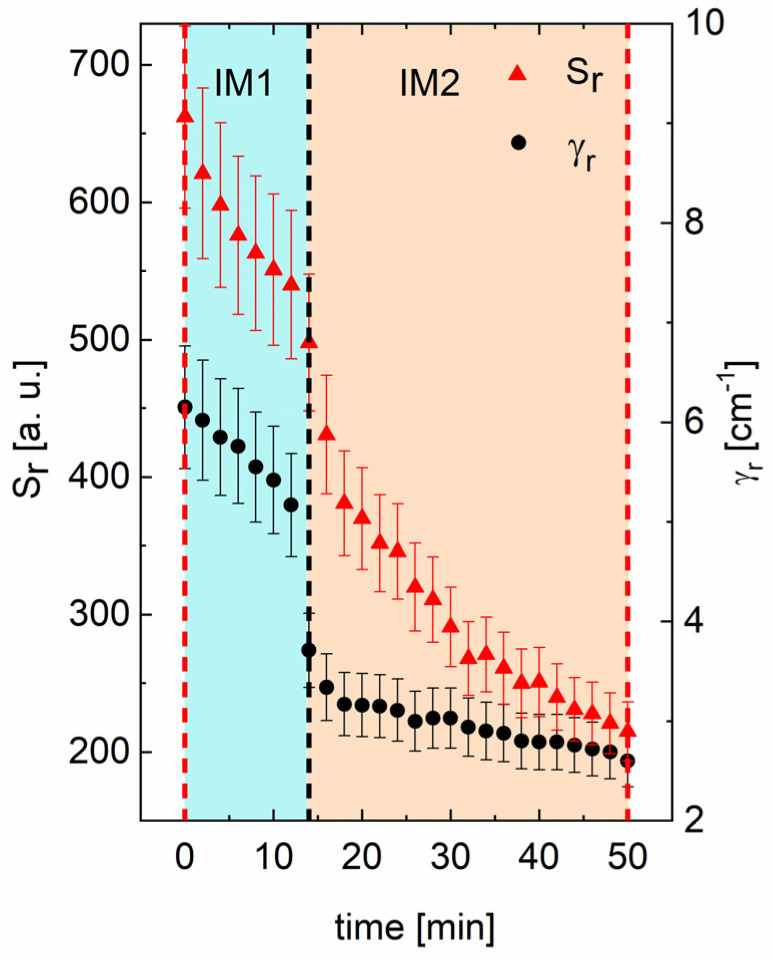
Strength *S_r_* (triangles) and relaxation rate *γ_r_* (circles) of the central peak as a function of time for PbZrO_3_:0.1Nb crystal at 233 °C. The black dashed line corresponds to the isothermal phase transition. The red dashed lines correspond to the measurement time marked with the red line at 233 °C in Figure 4.

The time-dependent changes in strength and relaxation factor of the quasi-elastic part of Raman scattering fitted to a single Debye-type function are represented in Figure 7. This part of scattering is related to the central peak (CP), which—in materials with the structure of ABO_3_ perovskite containing lead—dramatically influences the dielectric divergence at T_C_ and is commonly associated with the relaxational movement of lead ions. It is worth underlying the reduction of the CP parameters in Figure 7 occurs in a similar time (about 14 min) as in which evolution of Raman line frequencies. Hence, we state that the isothermal transition is connected with lead sublattice behaviour. Proof of that is that the dielectric strength *S_r_* calculated from the CP is the same as low-frequency permittivity measured at the same temperature (see Figure 1b in [23]. In addition to the lead-related vibrations, we should not wholly ignore central ion-related vibrations. Recently, such kind of relaxation of Ti towards [111] direction has been found in barium titanate, below and above T_C_ [43]. It has been linked with polar region appearance than with isothermal transformation.

After about 50 min, the sample cooling was resumed. At temperature 227 °C, a transition to the AFE phase was detected, at which additional modes, compared to those present in the IM2 phase, evolve. These observations are in line with previous studies reported in [23].

## 4. Discussion

In perovskites containing lead, such as antiferroelectric PbZrO_3_, the co-existence of different dynamics due to strongly anharmonic optic–acoustic mode coupling decides the transition at T_C_. The antiferroelectric transition in PbZrO_3_ is attributed to softening of several longitudinal and transverse zone boundary modes [15]. The longitudinal modes refer to the Pb sublattice and the transverse to the oxygen octahedral rotations [44]. It was shown that this coupling leads to clusters’ appearance with increasing length scale when T_C_ is approached. In this increasing length scale, the dynamic of the elastic properties is dominant. In the case of PbZrO_3_ single-crystals doped with Nb, the similar sublattice instabilities decide the transition at T_C_ and between intermediate IM1 and IM2 phases [22]. This paper showed that a similar transition could also be realized via an isothermal process. The isothermal phase transition across IM1–IM2 phases was discovered independently in two different compositions of PbZrO_3_ single crystals with Nb dopant (i.e., 0.077 and 0.1 mol% of Nb) and two different techniques_._ Based on that, we claim that the observed isothermal phase transition is an intrinsic phenomenon.

To understand this phenomenon, we have to answer the question of the driving force for that isothermal transition. The simplest explanation would be that it is connected with the time needed for the crystal to reach the thermodynamic equilibrium. However, the crystals were of small sizes, so we ignore such an explanation. Moreover, the temperature at which the isothermal transition was observed to be far enough from the transition temperature point −Tc.

The IM1 phase has been recognized as a phase in which instabilities in Pb-related lattice vibrations are responsible for the IM2 phase appearance. This transformation occurs either by lowering the temperature or over time, provided that time is long enough, i.e., dozens of minutes. Such a long time is certainly unexpected. We guess it is connected with the ordering process in the sublattice of Pb ions, which prefer to be coupled in pairs, as it is finally achieved in the pure antiferroelectric phase. In this ordering process, thermal fluctuations are the main actor. However, the essential question is why this thermal activation has to last so long to induce transformation. This is connected with the long-time evolution in Pb sublattice from the short to long-range order. This long waiting for transition may be related to the presence of two different central ions, Zr and Nb. For example, a low concentration of Nb causes clusters to form and disrupts the order of the lead sublattice. The same would concern the isothermal transition observed in PZO doped with a small amount of Ti [45,46,47].

Discussing the mechanism of isothermal transition domain boundaries in which local stresses and polar orders need to relax in time has to be considered. The fact that the time after which isothermal transition begins is shorter at higher temperatures convinces us that only thermal fluctuations that activate ordering in Pb sublattice are the driving force in this intricate phenomenon. This is because the structure of domains and thus domains boundaries are stable, and do not evaluate in phase IM1 before the transition to phase IM2.

Regardless of the driving force nature which influences the isothermal transition, it is essential to remember that in the case of a phase in which the co-existence of phases is observed, all experiments have to be conducted with special care. The idea is to obtain samples in well-defined thermodynamic and structural states.

## 5. Conclusions

The above-described research proves that the isothermal phase transition occurs in PZO:Nb single crystals. It is treated like a process of self-organization of the structure at a constant temperature and has an explicit long-term relaxing nature.

Discontinuity of the strength and relaxation rate of the central peak observed in Raman light scattering spectra were a function of time for niobium doped PZ single crystals. In materials with the structure of ABO_3_ perovskite, this central peak is related to lead and dramatically influences the dielectric divergence at T_C_. Proof of that is that the dielectric strength *S_r_* calculated from the central peak is in the same order as low-frequency permittivity measured at the same temperature. Thus, we state that the isothermal transition is connected mainly with the lead sublattice. The thermal fluctuations, which activate ordering in the Pb sublattice, drive this complex phenomenon.

## Data Availability

The data presented in this study are available on request from the corresponding author.
